# A Case of Drug-Induced Immune Thrombocytopenia Caused by Haloperidol

**DOI:** 10.7759/cureus.91687

**Published:** 2025-09-05

**Authors:** Yoshihiko Kanno, Yoshinao Ono, Katsuya Takita

**Affiliations:** 1 Respiratory Medicine, Kesennuma City Hospital, Kesennuma, JPN; 2 Respiratory Medicine, Tohoku University Graduate School of Medicine, Sendai, JPN

**Keywords:** antipsychotic drugs, delirium, drug-induced immune thrombocytopenia, haloperidol, platelet transfusion

## Abstract

Drug-induced immune thrombocytopenia (DITP) is a rare but potentially fatal condition caused by immune responses to certain drugs, leading to platelet destruction or impaired production. Commonly implicated drugs include quinine, sulfamethoxazole, penicillin, and linezolid. Haloperidol is an extremely rare causative agent, with only two previous cases reported. This case highlights a rapid onset of DITP following haloperidol administration. Clinicians should remain aware that haloperidol may be a potential cause of drug-induced thrombocytopenia, including rare thrombotic thrombocytopenia. Prompt recognition and timely intervention are critical for ensuring positive clinical outcomes in affected patients.

## Introduction

Drug-induced immune thrombocytopenia (DITP) is a rare but potentially fatal condition, occurring at an estimated rate of 10 cases per million annually, with a reported mortality rate of 1.5％-6.2％ in recent epidemiologic studies and systematic reviews [1-3]. It results from humoral immune responses against platelet antigens, leading to increased platelet destruction, consumption, or reduced production [4]. Symptoms typically appear within one to 10 days of drug exposure, with nadirs seen between four hours and 10 days [5], resolving within five to seven days after discontinuation [6]. While commonly caused by quinine, sulfamethoxazole, and penicillin [7], DITP from haloperidol is extremely rare, with only two prior cases reported [8,9]. Haloperidol is among the most commonly utilized agents for the management of delirium, alongside other antipsychotics such as risperidone and quetiapine. Delirium is a frequent neuropsychiatric syndrome observed in hospitalized older adults, with reported prevalence rates ranging from 19％ to 55％ [10,11]. Advanced age represents a significant risk factor for the development of delirium, highlighting the critical importance of its effective management [12]. This report highlights a rare, rapid-onset haloperidol-induced DITP.

## Case presentation

**Figure 1:**
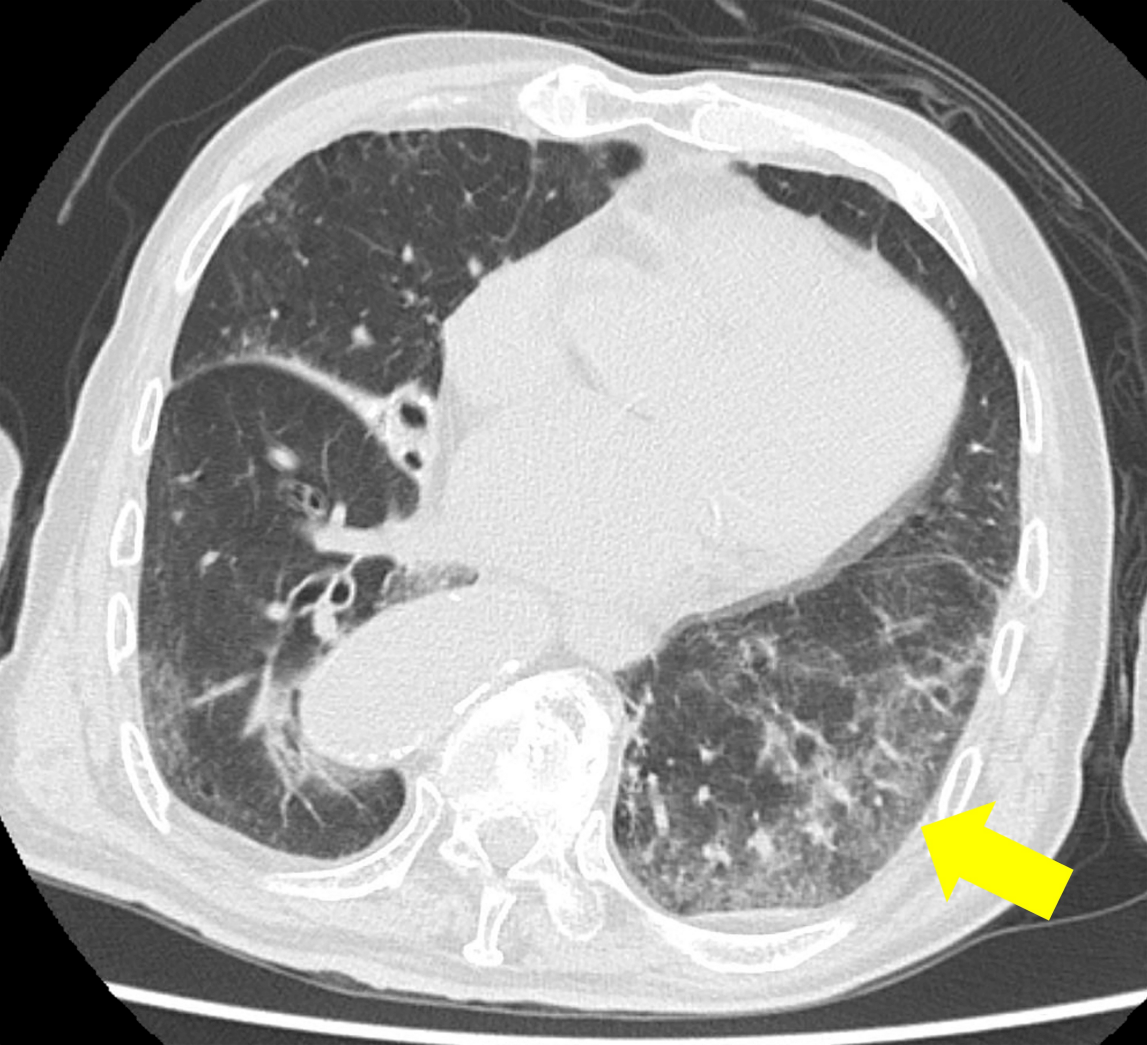
Computed tomography (CT) of the chest. CT showing ground-glass opacity in the left lower lobe (yellow arrow). CT findings were not directly related to the subsequent development or diagnosis of drug-induced immune thrombocytopenia (DITP).

A 93-year-old male presented to the emergency department with a chief complaint of fever. His initial vital signs included a body temperature of 38.4 ℃, blood pressure of 130/84 mmHg, oxygen saturation (SpO₂) of 91%, and a respiratory rate of 12 breaths per minute. Chest computed tomography (CT) revealed ground-glass opacity in the left lower lobe (Figure 1), consistent with a diagnosis of aspiration pneumonia.

Laboratory evaluation revealed an elevated inflammatory response without other significant abnormalities (Table 1).

**Table 1:** Laboratory data on admission. WBC: white blood cells, Neut: neutrophils, Lymph: lymphocytes, Mono: monocytes, Eosin: eosinophils, Baso: basophils, RBC: red blood cells, Hb: hemoglobin, Ht: hematocrit, Plt: platelets, TP: total protein, Alb: albumin, T-Bil: total bilirubin, AST: aspartate aminotransferase, ALT: alanine transaminase, LDH: lactate dehydrogenase, ALP: alkaline phosphatase, BUN: blood urea nitrogen, Cr: creatinine, CK: creatine kinase, CRP: C-reactive protein

Parameters	unit	Result	Reference range
WBC	/μL	6,500	4000-9000
Neut.	%	64.1	37.0-74.0
Lymph.	%	24.6	21.0-51.0
Mono.	%	6.4	2.0-10.0
Eosin.	%	4.2	0.0-7.7
Baso.	%	0.7	0.0-2.0
RBC	×10^4^ /μL	442	427-570
Hb	g/dL	12.7	14.0-18.0
Ht	%	40	40.0-52.0
Plt	×10^4^ /μL	17.4	15.0-35.0
TP	g/dL	6.2	6.5-8.2
Alb	g/dL	3.7	3.8-5.3
T-Bil	mg/dL	1.7	0.3-1.2
AST	U/L	22	8-38
ALT	U/L	12	5-40
LDH	U/L	288	124-222
ALP	U/L	84	38-113
BUN	U/L	18.6	8.0-20.0
Cr	mg/dL	0.91	0.65-1.06
CK	mg/dL	56	62-230
Na	mEq/L	144	136-145
K	mEq/L	4.3	3.5-4.8
Cl	mEq/L	108	100-110
Ca	mg/dL	8.7	8.8-10.1
CRP	mg/dL	6.95	0.0-0.3

The patient was admitted for inpatient treatment.

Intravenous administration of ampicillin-sulbactam resulted in a prompt resolution of fever and reduction of inflammatory markers. Antibiotic therapy was discontinued on hospital day seven (with day one defined as the date of acute admission in this report). Due to impaired swallowing function, oral intake was deemed challenging, and transfer to a convalescent hospital was planned.

From admission, the patient exhibited agitation, which was interpreted as hyperkinetic delirium. In this case, the diagnosis of delirium was based on clinical assessment without the use of a standardized scale. Although non-pharmacological interventions such as rehabilitation and the use of a clock and calendar initially led to some improvement, his nocturnal delirium worsened on day 21. Since the symptoms persisted despite non-pharmacological interventions, a one-time intravenous dose of haloperidol (5 mg) was administered.

On day 22, the patient developed anal bleeding and hematuria. By day 23, a complete blood count revealed severe thrombocytopenia (platelet count: undetectable (0.0) /μL). There was no administration of heparin during hospitalization, and microscopic examination of the peripheral blood smear immediately after collection showed no evidence of platelet aggregation. Coagulation parameters remained normal, and there was no fever or rise in inflammatory markers. Autoantibodies, including antinuclear antibodies (ANA), anti-DNA, and anti-platelet antibodies, were negative. ADAMTS13 activity was normal.

**Figure 2:**
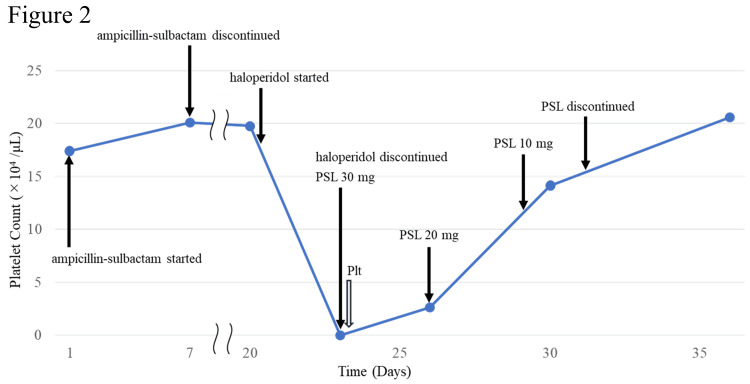
Platelet trend during hospitalization. Day 1 marks admission to the acute care hospital. Ampicillin-sulbactam was administered from day 1 to day 7. Haloperidol was given on day 21. For visual clarity, the distance between day 8 and day 19 has been shortened; day annotations correspond to days since acute admittance. The time course of platelet count. Plt: 20 unit of platelets transfused, PSL: prednisolone.

DITP was suspected due to haloperidol, so haloperidol was discontinued, and 20 units of platelets were transfused. Since idiopathic thrombocytopenic purpura (ITP) could not be fully excluded, prednisolone 30 mg was initiated. However, given the strong suspicion of DITP based on the clinical course, prednisolone was tapered by 10 mg every three days and discontinued by day 31. The platelet count improved to 14.1 × 10⁴ /μL on day 30. After steroid cessation, the platelet count remained stable, measuring 20.6 × 10⁴ /μL on day 36 (Figure 2).

In this case, the platelet count increased significantly after a short course of steroids, which is unlikely to occur in ITP, as ITP typically requires treatment with higher doses and longer duration of steroids to achieve and maintain remission. The prompt recovery following steroid taper strongly supports a diagnosis of DITP rather than ITP. The patient was then transferred to a convalescent hospital, where follow-up evaluations showed sustained platelet stability: 19.5 × 10⁴ /μL at 30 days post-transfer and 20.7 × 10⁴ /μL at 60 days.

## Discussion

This case report describes DITP caused by haloperidol. The annual incidence of DITP is estimated to be approximately 10 cases per million population, with higher rates observed in hospitalized patients and the elderly, according to epidemiological studies conducted in the United States and Europe [1]. Although the exact mechanisms underlying DITP remain unclear, it is believed to involve platelet-reactive IgG and IgM antibodies that target glycoprotein IIb/IIIa and Ib/V/IX complexes on platelet surfaces in the presence of specific drugs [13,14].

DITP is a diagnosis of exclusion and requires heparin-induced thrombocytopenia (HIT), ITP, thrombotic thrombocytopenia purpura (TTP), and pseudothrombocytopenia to be ruled out. Additionally, there were no schistocytes observed on peripheral smear, and ADAMTS13 activity was normal, ruling out TTP. The rapid recovery of platelet count after discontinuation of haloperidol and its inconsistency with the typical clinical course of ITP further supported the diagnosis of DITP.

George et al. proposed four criteria for diagnosing DITP: (i) thrombocytopenia onset after drug exposure and resolution after its discontinuation, (ii) no alternative explanations for thrombocytopenia or resolution upon rechallenge with other agents, (iii) exclusion of other thrombocytopenic conditions, and (iv) recurrence of thrombocytopenia following re-exposure to the offending drug [6]. However, these criteria include prospective testing, which is often impractical in clinical settings. In contrast, Arnold et al. suggested alternative criteria that exclude prospective testing and focus on clinical observations, including: (i) Severity: Platelet nadir typically <20 × 10⁹/L, (ii) clinical signs: Presence of bleeding complications, (iii) onset timing: Occurs five to 10 days after initial exposure, or within hours of the first drug exposure, or exposure to a drug taken in the past, (iv) drug exposure history: Documentation of drugs associated with DITP [7]. In this case, re-exposure to haloperidol was not feasible. Although the case was categorized as “probable” under George’s criteria, it fulfilled Arnold’s criteria, leading to a definitive diagnosis of DITP.

Management of DITP requires prompt discontinuation of the inciting drug. Platelet transfusion should be initiated if there are clinical signs of bleeding, or with platelet count <10,000 /μL [15]. In this case too, the platelet count was extremely low, so a platelet transfusion was administered. At first, it was not possible to distinguish between ITP and this case, so prednisolone was used in combination, but since the course of the disease ruled out ITP, the steroids were quickly terminated.

DITP caused by haloperidol has only been reported in two cases in the past [8,9]. One case developed after repeated administration of chlorpromazine, haloperidol, and thiothixene, and another case, it is thought that the toxicity of haloperidol was enhanced by the concomitant use of levothyroxine. In addition, in a prospective study comparing the incidence of thrombocytopenia in patients treated with haloperidol, chlorpromazine, fluphenazine decanoate and no drug therapy [16], there was no significant decrease in the haloperidol group, and it is thought that DITP due to haloperidol monotherapy is very rare.

When using antipsychotic drugs in the elderly, DITP should be kept in mind when a decrease in platelet count is observed.

## Conclusions

To our knowledge, no previous cases have been reported with haloperidol monotherapy. This case highlights the importance of considering drug-induced thrombocytopenia in patients who develop severe thrombocytopenia during the clinical course, especially in the presence of recent exposure to the culprit medication. Since DITP can lead to severe complications, including death, greater awareness of this syndrome is warranted, and early recognition with prompt treatment initiation is essential.
